# Drought coping strategies in cotton: increased crop per drop

**DOI:** 10.1111/pbi.12688

**Published:** 2017-02-20

**Authors:** Abid Ullah, Heng Sun, Xiyan Yang, Xianlong Zhang

**Affiliations:** ^1^ National Key Laboratory of Crop Genetic Improvement Huazhong Agricultural University Wuhan Hubei China

**Keywords:** ABA, cotton, drought stress, MAPK, ROS

## Abstract

The growth and yield of many crops, including cotton, are affected by water deficit. Cotton has evolved drought specific as well as general morpho‐physiological, biochemical and molecular responses to drought stress, which are discussed in this review. The key physiological responses against drought stress in cotton, including stomata closing, root development, cellular adaptations, photosynthesis, abscisic acid (ABA) and jasmonic acid (JA) production and reactive oxygen species (ROS) scavenging, have been identified by researchers. Drought stress induces the expression of stress‐related transcription factors and genes, such as ROS scavenging, ABA or mitogen‐activated protein kinases (MAPK) signalling genes, which activate various drought‐related pathways to induce tolerance in the plant. It is crucial to elucidate and induce drought‐tolerant traits via quantitative trait loci (QTL) analysis, transgenic approaches and exogenous application of substances. The current review article highlights the natural as well as engineered drought tolerance strategies in cotton.

## Introduction

**Figure 1:**
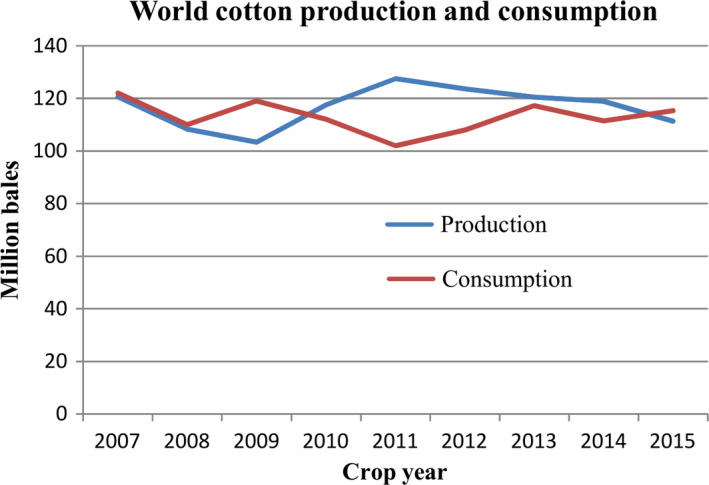
Unstable world cotton production and their consumption since 2007.

Cotton is grown as a leading commercial crop in more than 30 countries of world with major shares from China, India, the United States and Pakistan, and is predominantly cultivated in warmer regions (Riaz *et al*., 2013). According to statistics, China, India, the United State, Pakistan and Brazil were the top 5 cotton‐producing countries in 2014–2015, generating 6.5 M, 5.4 M, 3.5 M, 2.3 M and 1.5 M tones, respectively (Statista, 2015). As a glycophyte, cotton shows higher tolerance to abiotic stresses than other major crops. However, extreme environmental conditions, such as drought affect growth, productivity, and fibre quality of cotton (Parida *et al*., 2007). According to a press release from the United States Department of Agriculture (USDA), cotton production is expected to decline due to drought stress (USDA, 2015). Similarly in Pakistan, cotton production declined by 34% to just 9.68 M bales against the production of 14.4 M bales from previous year because of drought and high temperature (Dawn news, 2016). In addition to cotton, other crops were also affected by drought, as approximately 67% of crop losses were due to drought stress over the last 50 years in the United States (Comas *et al*., 2013). The impacts of drought on cotton are widespread and varied, which makes it difficult to determine accurate financial estimates (Table 1). As shown in Figure 1, world cotton production was very low in 2008 and 2009, which led to a significant decrease in stocks in 2009. Therefore, cotton prices were increased in 2010 and 2011, resulting in the cotton consumption decline of 10% in 2011. Cotton production was higher than demand from 2010 to 2013; however, the production decreased from 2011, with a significant decrease of 6.5% in 2015 from 2014, while consumption is increasing by approximately 6.5 million bales annually (Figure 1). Thus, we need to establish policies for the production and consumption of the cotton. Moreover, it is also necessary to produce stress‐tolerant varieties of cotton due to the uncertain conditions in the future. On the other hand, the emphasis should not be only on stress‐tolerant variety of cotton, although plant survival is very critical in the early stages of growth, stress‐tolerant variety should therefore be based on stability of yield. It is known that improving of yield and maintaining yield stability of cotton crop, under normal as well drought stress conditions, is essential for the growing global population.

**Table 1:** Direct and indirect impacts of drought on cotton and its management

Direct impacts	Indirect impacts	Management
Damage plants systems	Food scarcity	Drought‐tolerant varieties should develop
Reduce crop productivity	Reduce income of farmers and agribusiness	Effective impact assessment procedures should develop
Reduce water level	Increase prices of foods and goods	Pro‐active risk management measures
Increase insect infestation	Increase unemployment (companies dealing with agriculture will stop working)	Make plans aimed at increasing the coping capacity
Increase plant diseases	Increase crime and insecurity	Efficient emergency response programs should be planned which can be used for reducing the impacts of drought
	Cause pollution in the concern area	Meetings should conduct on national and international level about drought stress
	Migration	Early warning system should develop to make decision earlier

Despite the complexity of drought tolerance mechanism in cotton, tremendous progress has been made in understanding the drought tolerance mechanism. Morpho‐physiological, biochemical and molecular adaptations by nature or by genetic engineering can lead to the drought‐tolerant variety of cotton. The current review discusses effective techniques to alleviate the negative effects of drought stress in cotton and maintain the productivity as well as fibre quality. Moreover, the mechanisms of drought tolerance in cotton and strategies to induce tolerance to drought are also discussed.

## Morpho‐physiological mechanism of cotton in responses to drought stress

**Figure 2:**
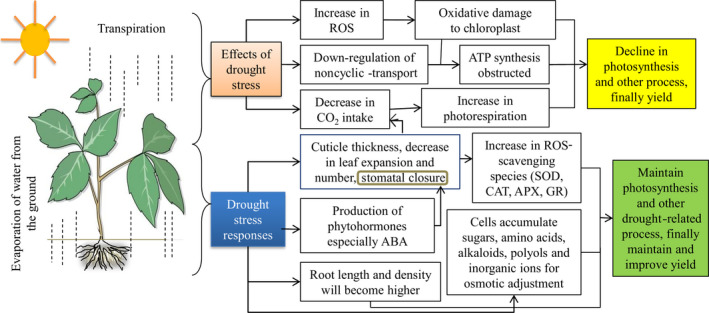
Numerous effects of drought stress on cotton and their responses.

Drought stress causes a wide range of morpho‐physiological and biochemical changes that adversely affect the development as well as the productivity of the cotton (Figure 2). Generally, drought stress severely restricts cotton growth and development, such as affecting plant height, leaf dry weight, stem dry weight, leaf area index, node number, fibre quality, canopy and root development (Loka *et al*., 2011). Specifically, net photosynthetic rate, transpiration rate, stomata conductance, carboxylation efficiency and water potential of cotton leaves decrease significantly during drought conditions (Kumar *et al*., 2001). Recently, Hejnák *et al*. (2015) studied the detrimental effects of drought stress on cotton. According to their results, 50% dry matter accumulation of *Gossypium barbadense* (*G. barbadense*) was limited under drought stress. Moreover, the stomata conductance, photosynthetic rate and transpiration rate were also decreased under water deficit. Like other plants, cotton has acquired a wide range of morpho‐physiological, biochemical and molecular mechanisms in response to multiple stresses that enable them to avoid and/or tolerate these stress factors and survive in harsh environments.

The plant drought tolerance mechanisms can be divided into four strategies: drought avoidance, drought escape, drought tolerance and drought recovery (Fang and Xiong, 2015). Drought avoidance and drought tolerance are the two major strategies of plants against drought stress. Drought avoidance is the maintenance of key physiological processes, such as stomata regulation, root system development and others, during moderate drought conditions. Drought tolerance is the capability of plants to withstand severe dehydration through specific physiological activities, such as osmotic adjustment via osmoprotectants (Luo, 2010). Drought escape is the ability of plants to adjust their growth period or lifecycle, such as the cotton variety with a short life cycle, to avoid the seasonal drought stress (Manavalan *et al*., 2009). Drought recovery of plants is the capability to resume growth and yield after exposure to severe drought stress. Cotton has evolved several common morpho‐physiological strategies against drought stress, which have been discussed in this section, such as stomata regulation, root development, photosynthetic response and osmotic adjustment.

### Stomata regulation

Reduction of water loss through leaves is a crucial phenomenon in cotton plants under drought stress. Wilting and rolling of leaves result in less radiation and thus reduced water loss (Fang and Xiong, 2015). Plants often show various xeromorphic characters and have structures that promote drought tolerance, such as thicker and smaller leaves, a thicker cuticle epidermis, more epidermal trichomes, thicker palisade tissues, smaller and denser stomata, a high ratio of palisades to spongy parenchyma thickness, and a developed vascular bundle sheath (Hetherington and Woodward, 2003; Iqbal *et al*., 2013). For example, the cotton variety, *Gossypium hirsutum* (*G. hirsutum*) YZ1, has smaller leaves as compared to *G. hirsutum* Y668. Stomata regulation plays a pivotal role in gas exchange between tissues and the atmosphere. It is one of the key mechanisms that allow plants to produce energy and maintain cellular function. Ninety per cent of water losses (transpiration) from plants occur though stomata openings (Wang *et al*., 2009). In cotton, closure of the stomata is the first step to reduce water loss during drought conditions, when the rate of transpiration is very high. Stomata conductance could be a potential indicator of drought tolerance in cotton as there is a negative correlation between drought tolerance and stomata conductance.

### Root development

All root traits are potentially important in the drought stress; however, hydraulic conductance and plant allometry have been of particular interest to researchers. Various scientists have reviewed the potential function of roots under drought stress (Comas *et al*., 2013). More profuse (higher root length density) and deeper root systems in the soil are often proposed as desirable characteristics for drought adaptation. In a case, Luo *et al*. (2016) reported that mild and initial‐stage drought stress enhanced root length in cotton, but long‐time water deficit reduced the root activity as compared to control plants. In another study, transgenic cotton plants were more tolerant to drought stress, with a better root system than in wild type (Liu *et al*., 2014). Similarly, the transgenic cotton plants harboured *Arabidopsis* that enhanced drought tolerance 1/homodomain glabrous 11 (*AtEDT1/HDG11*) gene had well‐developed roots in addition to other drought‐tolerant features (Yu *et al*., 2015).

### Photosynthesis

Drought stress causes stomata closure, which leads to the decreased CO_2_ intake, affecting the rate of photosynthesis and consequently reduces growth and yield (Chaves *et al*., 2009). However, in some cases, stomata conductance is not always associated with the rate of photosynthesis, but this still needs to be elucidated (Von Caemmerer *et al*., 2004; Xu *et al*., 2010). Photosynthesis is severely affected along with growth as the water deficit increases gradually in the field of cotton. For example, it was found that photosynthesis as well as transpiration was affected under drought conditions in cotton (Deeba *et al*., 2012; Li *et al*., 2012). Interestingly, it has been reported that young leaves of cotton are photosynthetically more tolerant to drought and heat as compared to mature leaves. When young leaves were subjected to high temperature (37 °C), no decline was observed in net photosynthesis. In contrast, mature leaf net photosynthesis declined 66% under the same conditions (Chastain *et al*., 2016). In another field study of cotton for two consecutive growing seasons, a decreased lint yield was observed as net photosynthesis declined under water‐deficit conditions in the first growing season. However, no change was observed in the yield of drought‐treated field due to high rainfall in the next growing season (Chastain *et al*., 2014). These studies revealed that drought stress reduces photosynthesis in cotton which in turn affects growth and yield.

### Osmotic adjustment

At the cellular level, water deficit affects turgidity and osmotic balance in the cell. Osmotic adjustment is a critical adaptation to reduce the effects of drought‐induced damage in crop plants. Plant defence mechanisms also include osmoprotectants or osmolytes that regulate homoeostasis following drought and salinity stress on a cellular level. Drought stress has negative effects on osmotic balance, and therefore, plants accumulate different organic and inorganic substances to reduce the osmotic potential in response to drought stress (Fang and Xiong, 2015). Numerous organic compounds, including amino acids (proline, glycine), sugars (trehalose, fructan), sugar alcohols (mannitol, sorbitol, D‐ononitol), amines and polyamines (polyamine, betaines), polyols, ectoine, alkaloids and inorganic ions, known as osmoprotectants/osmolytes, are involved in osmotic adjustment (Fang *et al*., 2015; Singh *et al*., 2015). These solutes assist in protecting proteins and membranes from the damage due to high concentrations of inorganic ions and oxidative damage under drought stress (Chen and Murata, 2011) and multiple stresses, such as drought and salinity (Khan *et al*., 2015). The exogenous application of osmoprotectants (proline and glycinebetaine) has been shown to be effective in reducing the harmful effects of drought stress in cotton (Noreen *et al*., 2013). The transgenic cotton plants with enhanced glycinebetaine accumulation were more tolerant to drought stress than control plants and had increased photosynthesis, higher relative water content, increased osmotic adjustment, lower lipid membrane peroxidation and a lower percentage of ion leakage (Lv *et al*., 2007). Ectopic expression of a mustard annexin gene, *AnnBj1*, enhanced proline content and sucrose, which increased drought tolerance in cotton (Divya *et al*., 2010). Moreover, overexpression of *GhAnn1,* a cotton annexin gene, enhanced the tolerance to drought and salt by increasing the activity of superoxide dismutase (SOD) and elevated levels of proline and soluble sugars (Zhang *et al*., 2015).

## Biochemical and molecular mechanism of drought tolerance in cotton

**Figure 3:**
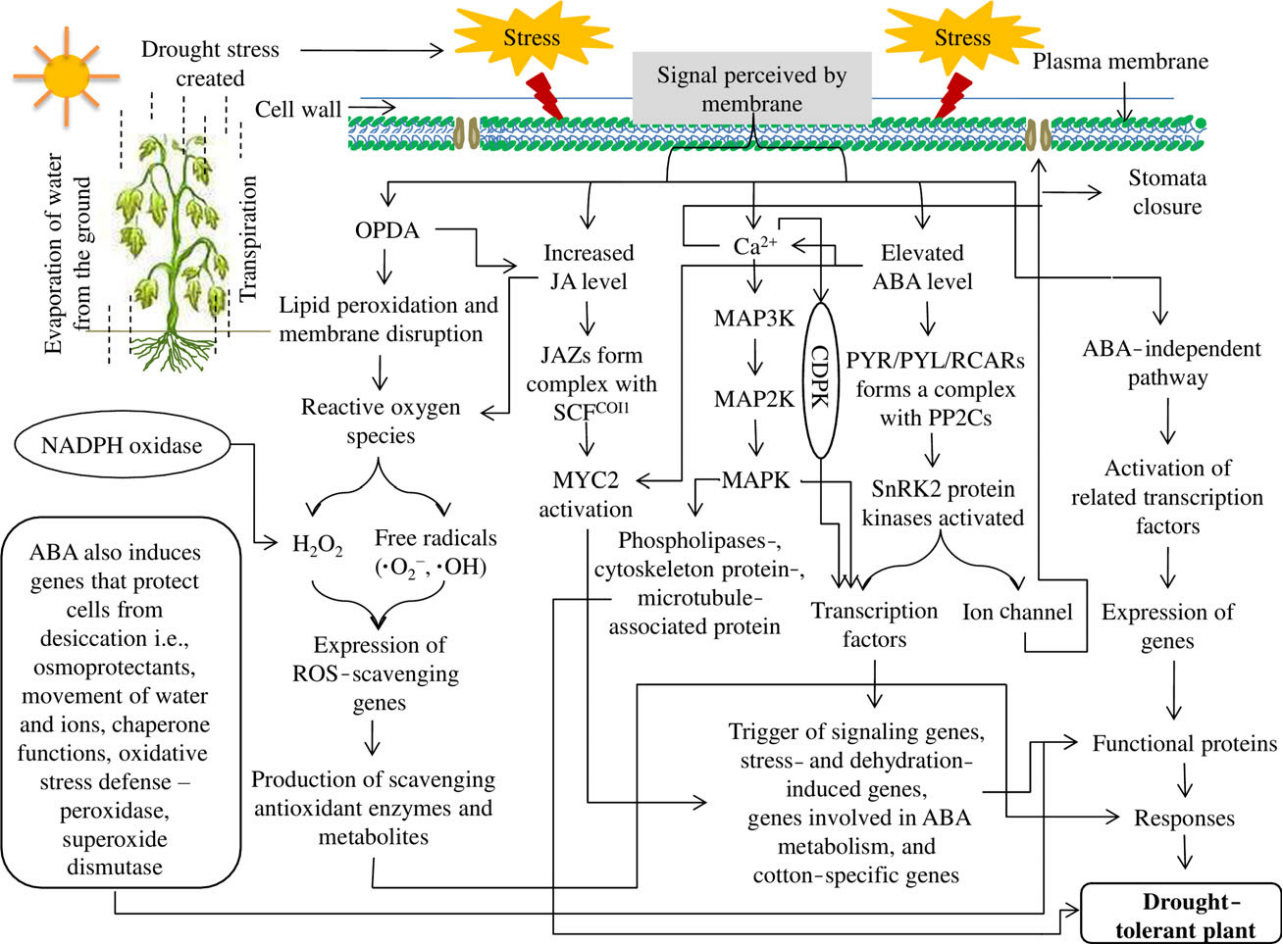
Various signalling pathways connectively enhance drought tolerance in cotton. These pathways work together to maintain their normal activities under drought stress.

Similar to animals, plants also have a defence mechanism through which they respond to various biotic and abiotic stresses. The drought tolerance mechanism is very complex because it is a multigenic system that is related to various morpho‐physiological, biochemical and molecular processes (Figure 3). Other than morpho‐physiological mechanism, cotton has evolved several signal transduction pathways in response to drought stress.

### Abscisic acid (ABA)

**Figure 4:**
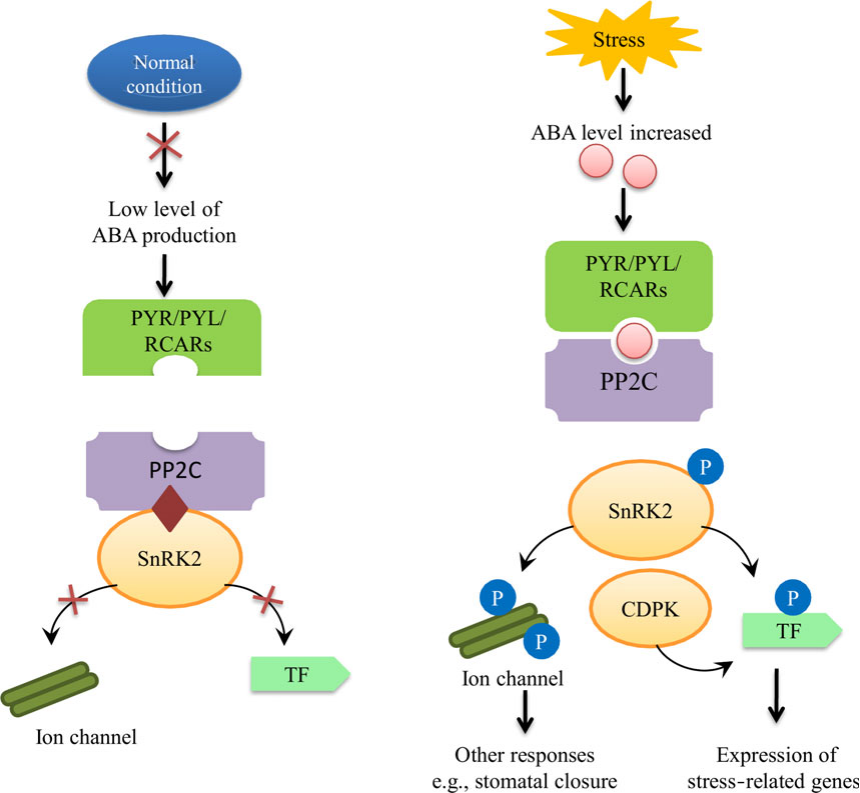
ABA mediated signalling pathway during normal and stress conditions. Under normal conditions, ABA content is low, and SnRK2 protein kinase activity is inhibited by PP2C phosphatases. Under drought stress, the cellular ABA level increases, and ABA then binds to PYR/PYL/RCARs, which in turn bind and inactivate PP2Cs. The SnRK2s autoactivate when they dissociate from PP2Cs. Activated SnRK2s phosphorylate downstream targets and trigger ABA‐induced physiological and molecular responses.

ABA is one of the most important stress hormones and participates in various crucial physiological processes during the plant life cycle, including stress responses, development and reproduction. Studies indicate that osmotic stress occurs due to high drought conditions or salt stress or when water availability is reduced through water loss and turgor pressure (Boudsocq and Lauriere, 2005). Osmotic stress promotes the synthesis of ABA, which activates gene expression and adaptive physiological changes (Yamaguchi‐Shinozaki and Shinozaki, 2006). After stress signal perception by the plasma membrane, ABA synthesis is initiated, which occurs mostly in the plastids, with the exception of xanthoxin conversion to ABA, which takes place in the cytoplasm (Seo and Koshiba, 2002). Generally, ABA synthesis occurs in the roots. It is then transported via vascular tissues, and it shows stomatal closure responses in a variety of cells, such as guard cells (Kuromori *et al*., 2010). As in other plants, perception and signal transduction of ABA in cotton are mediated by two pathways, which are ABA‐dependent and ABA‐independent. ABA‐dependent signalling pathways play a critical role in stress‐responsive gene expression during various stresses, especially osmotic stress. ABA receptors are important elements for ABA signal transduction. Various receptors have been identified in different subcellular compartments, including the plasma membrane, nucleus, cytosol and chloroplast envelope. Under normal conditions, ABA content is low, and sucrose nonfermenting 1‐related protein kinase 2 (SnRK2) protein activity is inhibited by protein phosphatase 2C (PP2C), which leads to dephosphorylation. When plants suffer drought stress, the cellular ABA level increases, and ABA then binds to PYR/PYL/RCARs, which in turn bind and inactivate PP2Cs. The SnRK2s are autoactivated when they dissociate from PP2Cs. Activated SnRK2s phosphorylate downstream targets and trigger ABA‐induced physiological and molecular responses (Danquah *et al*., 2014; Dong *et al*., 2015; Mehrotra *et al*., 2014; Yoshida *et al*., 2014)). ABA regulates many stress‐related genes to enhance drought tolerance in cotton plants (Figure 4). Overexpressing an ABA‐induced cotton gene *GhCBF3* in *Arabidopsis* enhanced drought and salinity tolerance in transgenic lines, with higher proline content, relative water content and chlorophyll content in transgenic lines than those in wild type. In the presence of ABA, stomatal aperture was smaller in transgenic lines, and expression level of *AREB1* and *AREB2* was remarkably higher than wild type. They suggested that *GhCBF3* enhance drought and salt tolerance via ABA signalling pathway (Ma *et al*., 2016).

### Jasmonic acid (JA)

Jasmonic acid (JA) is another phytohormone derived from α‐linolenic acid. JA and its active derivatives, which are known as jasmonates, have a significant role in regulating stress responses of plants to various biotic as well as abiotic stresses. In addition to plant growth and development, JA is also involved in root growth, fruit ripening, tendril coiling and viable pollen production (Wasternack, 2007). JA has been shown to participate in the response to drought conditions. Genomewide functional analyses of cotton were performed to analyse the molecular mechanism of drought resistance, and they identified various genes related to JA signalling pathways (Chen *et al*., 2013). Tan *et al*. (2012) reported that JA application inhibited fibre elongation in cotton. Similarly, several studies have also shown that exogenous application of jasmonates enhances plant resistance to water‐deficit conditions (Bandurska *et al*., 2003). Similar to ABA, various studies have shown that jasmonates also participate in the regulation of stomatal closure (Riemann *et al*., 2015).

Although JA signalling pathway has not been fully elucidated, its biosynthesis and signalling pathway have been reviewed extensively in the last few years (Ahmad *et al*., 2016; Kombrink, 2012; Wasternack and Hause, 2013). The jasmonate‐zim domain (JAZ) repressor proteins have a key role in the JA signalling pathway—they function as a switch for JA signalling. In normal conditions, when JA is absent, jasmonate‐insensitive/jasmonate‐zim (JAI3/JAZ) proteins bind to various transcription factors, including MYC2 (Myelocytomatosis), and limit their activity. However, during stress conditions, when JA and its derivatives are present, degradation of JAZ proteins occurs as described above, resulting in active transcription factors (MYC2) that up‐regulate genes involved in stress responses (Chini *et al*., 2007). The signalling pathway and JAZ protein interactions in this pathway have been comprehensively reviewed (Wager and Browse, 2012). Generally, plant hormones do not function in discrete pathways but rather depend on each other at different stages to control environmental as well as developmental pathways. This results in signal transduction that can assimilate various processes and respond to the stress in a complex way (Riemann *et al*., 2015). The jasmonates, similar to ABA signalling, act as a hub where different processes are initiated to appropriately respond to drought stress.

### Reactive oxygen species (ROS)

Partial reduction of atmospheric O_2_ leads to the production of ROS, also known as active oxygen species (AOS) or reactive oxygen intermediates (ROI). Cellular ROS basically consist of four forms, hydrogen peroxide (H_2_O_2_), the hydroxyl radical (HO•), superoxide anion radical ($O_{2}^{-}$) and singlet oxygen (^1^O_2_). Two of these forms are especially very reactive, that is HO• and ^1^O_2_. They can harm and oxidize various components of the cell, such as lipids, proteins, DNA and RNA. Eventually, they can result in cell death if the oxidation of cellular components is not controlled (Fang *et al*., 2015). Subcellular locations, such as the mitochondria, plasma membrane, cell wall, chloroplast and nucleus, are responsible for the production of ROS (Gill and Tuteja, 2010). Under drought stress, the production of these ROS increases in various ways. For example, a reduction in CO_2_ fixation leads to decreased NADP^+^ regeneration during the Calvin cycle, which will reduce the activity of the photosynthetic electron transport chain. Moreover, during drought conditions, there is excessive leakage of electrons to O_2_ by the Mehler reaction during photosynthesis (Carvalho, 2008). The Mehler reaction reduces O_2_ to $O_{2}^{-}$ by donation of an electron in photosystem I. $O_{2}^{-}$ can be converted to H_2_O_2_ by SOD which can be further converted to water by ascorbate peroxidase (Heber, 2002). However, it is difficult to evaluate the levels of ROS produced during the Mehler reaction compared to those generated through photorespiration. Drought conditions also enhance the photorespiratory pathway, particularly when RuBP oxygenation is high due to limited CO_2_ fixation. Noctor *et al*. (2002) found that approximately 70% of total H_2_O_2_ production occurs through photorespiration under drought stress.

**Figure 5:**
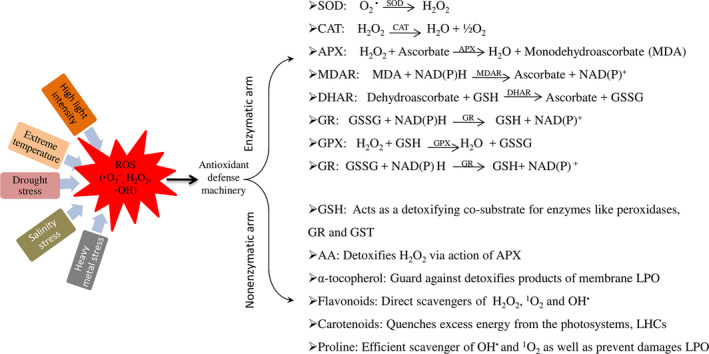
ROS scavenging machinery having two arms: enzymatic arm and nonenzymatic arm. Enzymatic arm contents on various enzymes which converting ROS into other substances. Likewise, Nonenzymatic arm content on other substances which scavenge ROS.

Plants have developed complicated scavenging mechanisms and regulatory pathways to monitor the ROS redox homoeostasis to prevent excess ROS in cells. Alterations in antioxidant enzyme metabolism could influence drought tolerance in cotton. The defence mechanism against ROS has been reviewed in detail by Das and Roychoudhury (2014). The antioxidant machinery has been developed by the plants to ensure survival (Figure 5). It has two arms, (i) enzymatic components, such as catalase (CAT), SOD, ascorbate peroxidase (APX), glutathione reductase (GR), guaiacol peroxidase (GPX), dehydroascorbate reductase (NADH) and monodehydroascorbate reductase (MDAR), and (ii) nonenzymatic antioxidants, such as reduced glutathione (GSH), ascorbic acid (AA), α‐tocopherol, flavonoids, carotenoids and the osmolyteproline (Figure 5). To scavenge ROS, these two arms/components work together (Das and Roychoudhury, 2014; Heiber *et al*., 2007; Wu *et al*., 2015). APX, along with MDAR, NADH and GR, removes H_2_O_2_ via the Halliwell–Asada pathway (Uzilday *et al*., 2012). APOX reduces H_2_O_2_ to water by oxidizing ascorbate to MDHA and thus plays a key role in the ascorbate–glutathione cycle (de Azvedo Neto *et al*., 2006). MDHA is then reduced to ascorbate by MDHAR. However, two molecules of MDHA can be nonenzymatically converted to MDHA and dehydroascorbate, which is further reduced to ascorbate via the NADH and GR cycle (Szalai *et al*., 2009). In this cycle, glutathione (GSH) is reduced by GR oxidation to oxidized glutathione at the expense of NADPH (nicotinamide adenine dinucleotide phosphate). Glutathione reductase activity increased during drought stress to keep oxidized and reduced glutathione ratios at adequate level (Chan *et al*., 2013). The balance between ROS production and antioxidative enzyme activities determines whether oxidative signalling and/or damage will occur (Zhang *et al*., 2014c). The antioxidative capability of different cotton cultivars determines the resistance capability to drought stress. The drought‐tolerant cultivar M‐503 has constitutively active antioxidative enzymes, including SOD, APX, CAT and POX, which decrease the oxidative stress induced by lipid peroxidation (Sekmen *et al*., 2014). In cotton, drought induced the production of ROS, but on the other hand, the APX and GR activities also increased and maintained the ROS scavenging process until the plant recovered from stress conditions (Ratnayaka *et al*., 2003). Supplemental Zn in cotton contributed to alleviating oxidative injuries under polyethylene glycol‐simulated (PEG) drought stress because it enhanced SOD, CAT, APX activities and the content of nonenzymatic antioxidants (Wu *et al*., 2015). In another example, Zhang *et al*. (2014c) conducted an experiment on the cotton cultivars: drought‐resistant (CCRI‐60) and drought‐sensitive (CCRI‐27). They found that the CCRI‐60 cultivar was drought tolerant due to increased root length and vigour, antioxidant enzyme activities and significantly increased GR activity and proline content. CCRI‐60 has the ability to scavenge free radicals and provides better protection compared to CCRI‐27; thus, it is more resistant to drought and has increased growth. Down‐regulation of *GbMYB5* in *G. barbadense* resulted in decreased antioxidant enzyme activities such as, SOD, peroxidase (POD), CAT and glutathione S‐transferase (GST), and increased oxidative stress under drought conditions (Chen *et al*., 2015a). These results show that cotton has numerous genes involved in the antioxidant enzyme‐related pathways that need to be explored in drought‐tolerant cultivars. Moreover, other factors are also involved in improving the antioxidant machinery of cotton plants, such as Zn, (Wu *et al*., 2015).

### MAPK signalling pathway

Plants have developed various adaptations to environmental stresses that function through a series of molecular networks consisting of stress perception, signal transduction and expression of specific stress‐related genes. The mitogen‐activated protein kinase (MAPK) cascade is one of the key strategies developed by plants against multiple biotic and abiotic stresses that participates in signal transduction of extracellular stimuli and regulates responses. MAPK pathway is a highly conserved central regulator of various processes, including developmental programs, hormonal responses, cell division and apoptosis, proliferation and stress responses. A MAPK cascade is minimally composed of at least three distinct protein kinases, that is MAPKKK, MAPKK and MAPK, which activate each other in a sequential manner via phosphorylation (Ichimura *et al*., 2002). An activated MAPKKK first phosphorylates two serine and/or threonine residues in a conserved S/T‐X3‐5‐S/T motif located within the activation loop of the MAPKK. The activated MAPKK in turn phosphorylates MAPK on threonine and tyrosine residues in the invariant T‐X‐Y motif in the activation loop, and then MAPK phosphorylate specific targets and modulate the activity of other kinases, transcription factors, phospholipases, cytoskeletal proteins and microtubule‐associated proteins, whose altered activities mediate an extensive range of response (Danquah *et al*., 2014; Nakagami *et al*., 2005; Popescu *et al*., 2009).

To date, many reports have confirmed that MAPKs are involved in plant signal transduction in response to abiotic stresses, such as drought, salinity, cold and oxidative stress, in *Arabidopsis* and rice (Ning *et al*., 2010; Shen *et al*., 2012; Teige *et al*., 2004; Xing *et al*., 2008, 2015). In recent years, several genes involved in the MAPK pathway response to abiotic stresses have been identified in cotton (Table 2). Transcriptome analysis revealed that MAPK components are activated by diverse abiotic stresses, such as ABA, cold, drought and pH treatments (Zhu *et al*., 2013). Twenty‐eight putative MAPK genes distributed on 11 chromosomes were identified in the *G. raimondii* genome by performing a bioinformatics homology search. These MAPK genes are classified into the four known A, B, C and D groups and have diverse functions (Zhang *et al*., 2014b). From the above analyses, we conclude that MAPK signalling pathways are involved in the response to multiple environmental stresses in cotton. However, there are no reports on improving plant stress tolerance by engineering MAPK cascades in cotton thus far. We recommend improving cotton stress tolerance by engineering MAPK cascades, and we believed that MKK1, MKK3, MPK6 etc. (Table 2) are effective candidates to improve the tolerance against abiotic stresses because it have been evaluated in *Arabidopsis* and tobacco.

**Table 2:** List of cotton MAPK genes engineered in other plants

Name	Induced by stress	Transgenic plant	Phenotype/Result	Interaction	References
*GhMKK3*	Drought	*N. benthamiana*	Enhanced drought tolerance	*GhMPK7* and *GhPIP1*	Wang *et al*. (2016)
*GhMAP3K40*	Low temperature, NaCl, PEG, H_2_O_2_	*N. benthamiana*	Enhanced drought and salt tolerance at the germination stage but reduced drought and oxidative stresses tolerance at the seedling stage	*GhMKK4*	Chen *et al*. (2015b)
*GhMPK4*	High salinity, osmotic stress	*A. thaliana*	Enhanced the sensitivity to salt, osmotic stresses and exogenous ABA	–	Wang *et al*. (2015)
*GhMKK4*	NaCl, mannitol, ABA	*N. benthamiana*	Had no significant effects on salt or drought tolerance	–	Li *et al*. (2014)
*GhMPK17*	NaCl, mannitol, ABA	*A. thaliana*	Enhanced plant tolerance to salt and osmotic stresses	–	Zhang *et al*. (2014a)
*GbMPK3*	NaCl, cold, heat, dehydration, oxidative stress	*N. benthamiana*	Enhanced drought and oxidative stress tolerance	–	Long *et al*. (2013)
*GhMPK6a*	Cold, NaCl, PEG	*N. benthamiana*	Reduced drought and salt tolerance	*GhMKK4*	Li *et al*. (2013)
*GhMKK1*	NaCl, drought, H_2_O_2_	*N. benthamiana*	Enhanced salt and drought tolerance	–	Lu *et al*. (2013)
*GhMKK5*	Low temperature, NaCl, Wounding	*N. benthamiana*	Reduced the tolerance to salt and drought stresses	–	Zhang *et al*. (2012)
*GhMPK2*	ABA, NaCl, PEG, dehydration	*N. tobacum*	Reduced sensitivity to ABA, enhanced drought and salt tolerance	–	Zhang *et al*. (2011)
*GhMPK6*	ABA, NaCl, drought stresses	*Arabidopsis* mutant atmkk1	Recovers the wild‐type phenotype of *atmkk1* mutant	–	Luo *et al*. (2011)
*GhMPK16*	Low and high temperatures, mannitol, NaCl	*Arabidopsis*	Reduced drought tolerance	–	Shi *et al*. (2011)

### Calcium signalling pathway

Calcium is a key regulator of various cellular and physiological processes in higher plants. In signal transduction pathways, Calcium (Ca^2+^) is a universal second messenger that regulates a variety of physiological processes in cotton plants. In addition to other stresses and hormones, drought and ABA are involved in changes of cytoplasmic Ca^2+^ concentration (Li *et al*., 2015). Plant cellular calcium signals are detected and transmitted by three major classes of Ca^2+^ sensor molecules: calmodulin (CaM) and CaM‐related proteins, calcium‐dependent protein kinase (CDPK) and calcineurin B‐like proteins (CBLs). Calmodulin is acidic calcium‐binding protein that contains four EF hand motifs (helix‐loop‐helix structural domains that coordinate with Ca^2+^ ions). When Ca^2+^ bind to EF motif, conformational transformation undergoes in CaM that either promotes its own catalytic activity or its interactions with downstream target proteins. As long as calcium sensor genes related to CaM and CaM‐related proteins have been studied for cotton fibre elongation (Cheng *et al*., 2016; Tang *et al*., 2014), there are no such reports found on drought stress in cotton. It has been noted that only a few CDPKs in cotton have been characterized extensively. *GhCPK1* was identified for the first time that has role in calcium signalling events associated with fibre elongation (Huang *et al*., 2008). Wang *et al*. (2012) opened a new door after sequencing the draft genome of *G. raimondii*. Last year, Li *et al*. (2015) identified 41 CDPKs gene from the *G. raimondii* genome. Their study revealed that *GhCDPK3*,* GhCDPK2*,* GhCDPK11*,* GhCDPK16*,* GhCDPK28*,* GhCDPK35* and *GhCDPK14* genes are involved in drought and salt stress. Further, they noted that these genes also respond to ABA. CBLs proteins are another group of calcium sensor which are specific to higher plants and play a significant role in decoding calcium transients by specifically interacting with and regulating a unique family of CBL‐interacting protein kinases (CIPKs). *GhCIPK6* was induced by drought, salt and ABA treatments. In addition, overexpression of *GhCIPK6* in *Arabidopsis* significantly enhanced tolerance to drought, salt and ABA stresses (He *et al*., 2013). These reports indicate that change in the Ca^2+^ concentration transduce Ca^2+^ signals via CaMs, CDPKs and CBLs, which phosphorylate downstream targets and subsequently respond to drought and other abiotic stresses.

### Stress‐related transcription factors

Transcription factors are master regulators of normal cellular processes as well as respond to biotic and abiotic stresses. Plants including cotton respond and/or adapt to various stresses, and transcriptional modulation is one of the most important ways that induce or repressed a number of genes in plants under biotic and abiotic stresses. Transcription factors play a significant role in the stress signalling, from the perception of drought to the stress‐responsive gene expression by interacting with *cis*‐acting elements present in the promoter region of numerous drought stress‐responsive genes in the signal transduction processes. In this way, transcription factors activate signalling cascade of entire network of drought stress‐responsive genes that operate together in inducing plant tolerance to drought and other abiotic stresses (Guo *et al*., 2016). As a model plant, more than 1500 transcription factors of the *Arabidopsis* genome are thought to be involved in stress‐responsive gene expression (Lata and Prasad, 2011).

To increase the tolerance in cotton against drought stress, transcription factors are excellent candidates for the plant scientists. Various transcription factors (such as MYB, WRKY, ERF, NAC, bZIP) are involved in normal development as well as in stress (drought) response (Table 3). These transcription factors have been cloned and proven useful tool for stress tolerance in cotton and/or in other plants. The genetic engineering of transcription factor genes could activate drought tolerance pathways and enhance drought tolerance in cotton. Recently, a bZIP transcription factor gene, *GhABF2,* has been reported to be involved in the drought and salt tolerance in *Arabidopsis* and cotton. The transcriptomic analysis revealed that *GhABF2*‐regulating genes related to ABA. Overexpressing *GhABF2* cotton increased SOD and CAT activities as compared to wild‐type plants. Moreover, overexpressed plants showed better results in the field and meanwhile its yield were recorded higher than wild‐type plants (Liang *et al*., 2016). In another case, an R2R3‐type MYB transcription factor gene, *GbMYB5*, positively involved in response to drought stress in cotton. Overexpressing *GbMYB5* tobacco reduced water loss by decreasing the stomatal size and showed hypersensitivity to ABA, and survival rate was higher after drought treatment. In addition, proline content and antioxidant enzymes were enhanced, while malondialdehyde (MDA) production was lower in transgenic lines than in wild‐type plants. Furthermore, the transcript level of drought‐responsive genes (*NCED3*,* BG*,* RD26*), antioxidant genes (*SOD*,* CAT*,* GST*) and polyamine biosynthesis genes (*ADC1*,* SAMDC*) were generally higher in *GbMYB*‐overexpressing tobacco (Chen *et al*., 2015a). Similarly, tobacco plants with ectopic‐expressing *GhWRKY41* gene showed higher antioxidant enzyme activity, enhanced stomatal closure and reduced MDA content. In addition, the expression of antioxidant genes was also up‐regulated in transgenic plants exposed to osmotic stress. These characteristics of transgenic plants enhanced plant tolerance to drought stress (Chu *et al*., 2015).

**Table 3:** Transcription factors in cotton playing important role in drought and other abiotic stresses

Genes encoding transcription factors	Expressing plant	Mode of expression	Environmental condition	Beneficial features under drought and other abiotic stress	Abiotic stress type	References
*GhABF2* (bZIP)	*A. thaliana* and *G. hirsutum*	Overexpressed and silenced	Greenhouse and field	Regulated genes related to ABA, Increased the activities of SOD and CAT	Drought and salt	Liang *et al*. (2016)
*GhNAC2*	*A. thaliana* and *G. hirsutum*	Overexpressed	Greenhouse	Higher root length,	Drought	Gunapati *et al*. (2016)
						
*GbMYB5*	*G. barbedensis* and *N. tobacum*	Overexpressed and silenced	Greenhouse	Reduced stomatal size, rate of its opening and water loss, while proline content and antioxidant enzymes increased	Drought and salt	Chen *et al*. (2015a)
*GhWRKY41*	*N. benthamiana*	Overexpressed	Greenhouse	Induced stomatal closure, higher antioxidant activity and lower malondialdehyde content	Drought and salt	Chu *et al*. (2015)
*GhWRKY17*	*N. benthamiana*	Overexpressed	Greenhouse	Impaired ABA‐induced stomatal closure, Reduced ABA level, decreased the expression of ROS scavenging genes, reduced proline content, elevated electrolyte leakage, and malondialdehyde	Drought and salt	Yan *et al*. (2014)
*GhNAC8‐GhNAC17*	*G. hirsutum*	Up‐regulation	Greenhouse	NA	Drought, salt, heat and Cold	Shah *et al*. (2013)
*GhERF1*	*G. hirsutum*	Up‐regulation	Greenhouse	Signal regulation during stress and ABA production	Drought, salt and Cold	Qiao *et al*. (2008)
*GhERF4*	*G. hirsutum*	Up‐regulation	Greenhouse	Signal regulation during stress and ABA production	Drought, salt and Cold	Jin and Liu (2008)

## Strategies to induce drought tolerance in cotton

To enhance plant tolerance as well as vigour against drought, alternative solutions must be developed. In this way, we can maintain crop yields under extreme environmental conditions to overcome economic losses. Improvements in cotton productivity are urgently needed, especially in the areas where water availability is scarce. In this regard, cotton crops that require less water but produce higher yields and better fibre quality will be highly desirable. Cotton characteristics should be site‐specific according to the environmental conditions of that area for instance, an area having less rainfall need drought‐tolerant variety of cotton but on other side, saline area need a salt‐tolerant variety of cotton. Along with traditional breeding, development in the field of biotechnology can produce transgenic cotton that performs better in current and future environmental conditions. However, exogenous application of particular substances, including growth regulators, specific osmoprotectants and required minerals, can enhance drought tolerance in otherwise susceptible plants. The aim was to produce more cotton yield per drop, and in this regard, crucial approaches are needed to identify and enhance drought‐tolerant traits, such as quantitative trait loci (QTL) analysis, transgenic approaches and exogenous application of substances.

### Marker‐assisted selection (MAS) based on drought‐related QTLs/genes

Various minor genes, that is polygenes, have stronger additive effects against drought tolerance than other biotic and abiotic stresses. Thus, the sections of DNA (locus) located on chromosomes carrying these genes are known as quantitative trait loci (QTL). Natural genetic variability of a crop can be utilized either via direct selection under stress environments whether simulated or natural or through QTL mapping (polygenes) and subsequent marker‐assisted selection (MAS) (Ashraf *et al*., 2008; Ashraf, 2010). QTL mapping allows us to determine the location, numbers, degree of phenotypic effects and gene action pattern (Vinh and Paterson, 2005). The role of polygenes has been extensively evaluated using traditional methods, but DNA markers as well as QTL mapping have made it possible and convenient to analyse complex traits (Ashraf, 2010). Biological and proteomics analyses have identified drought tolerance‐related QTLs and proteins in crop plants. Furthermore, these drought‐related QTLs and proteins can be used as markers in breeding programmes to develop drought‐tolerant genotypes.

In cotton, using F3 families derived from the cross of *G. barbadense* cv. F‐177 and *G. hirsutum* cv. Siv'on, a subset of 33 QTLs identified under water‐deficit conditions, that is five QTLs for different physiological traits, 11 for plant productivity and 17 for fiber quality. Most of these QTLs were located on chromosome c2, 6 and 14 (Saranga *et al*., 2001). Based on marker‐assisted selection, near‐isogenic lines were produced by exchanging QTL for drought‐ and some yield‐related traits between *G. barbadense* cv. F‐177 and *G. hirsutum* cv. Siv'on (Levi *et al*., 2009a,b). Moreover, metabolite and mineral analyses were conducted for these two species with QTLs for drought‐ and productivity‐related traits. The *G. hirsutum* cv. Siv'on showed higher levels of metabolites under drought and well‐water conditions compared to *G. barbadense* cv. F‐177. Under drought stress, Siv'on (*Gh*) had higher mineral and metabolite content and greater water use efficiency. Moreover, Siv'on also showed stable photosynthesis and a greater assimilation rate than F‐177 under drought conditions. For most of the studied traits, Siv'on showed a marked adaptation to drought (Levi *et al*., 2011). In another QTL study, five QTLs for osmotic potential (two QTLs were on chromosome c1, while rest of three were on c2, 6 and 25 each contained one), three for chlorophyll (two were on c2 and one on c14), 25 for leaf morphology and various others for yield and biotic stress were identified in cotton. QTLs for leaf morphology were distributed across the genome which is associated with leaf size and shape. Most notably, chromosome c15 contained six QTLs, c17 contained four, c6 contained three and c1 and c9 contained two QTLs each, while c2, c3, c4, c10, c12, c18, c22 and c25 all contained one QTL each (Said *et al*., 2013). In addition, 106 microsatellite markers were used to investigate 323 *G. hirsutum* germplasms, treated by drought and salt, and 15 markers were found related to drought tolerance. For the drought tolerance, 12 markers showed negative allele affects and the remaining markers showed positive allele effects (Jia *et al*., 2014). Likewise, a field study conducted for two consecutive years under water‐deficit and well‐water conditions, 11 physiological and morphological traits were recorded. As a result of QTL mapping, 67 and 35 QTLs were identified under water‐deficit and well‐water conditions, respectively. Most notably, chromosome c16, c9 and c2 contained 13, 12 and 7 QTLs, respectively (Zheng *et al*., 2016).

### Transgenic approach

Plants respond to multiple abiotic stress conditions at the molecular level by altering gene expression (up‐ or down‐regulation), which further regulates a number of proteins, and as a result, various biological functions are altered (Deeba *et al*., 2012). The regulation of genes involved in the stress response is one of the key factors in plants that cope with abiotic stresses and enhance tolerance against these conditions (Hozain *et al*., 2012). There are thousands of genes in plants, and a number of them are involved in drought stress. Different techniques were used to identify specific genes such as, the amplified fragment length polymorphism (AFLP) was used by Park *et al*. (2012), who identified several genes expressed under drought stress in cotton (*G. hirsutum* L.). In their study, heat‐shock protein‐related and ROS‐related transcripts were induced by water deficit. In another case, various stress‐related genes were identified by constructing normalized cDNA libraries of *G. barabadense* regarding drought‐, salt‐, heat‐, cold‐ and phosphorus‐deficit stresses (Zhou *et al*., 2016). It is possible to transfer specific traits or gene of interest, that is drought‐tolerant genes, from an organism of interest into another organism to obtain the desired characteristic by genetic engineering or biotechnology (Herdt, 2006). Recently, scientists transformed various drought‐tolerant genes into cotton, resulting in drought‐resistant plants (Table 4). Overexpressing of *TsVP*, an H^+^‐PPase gene from *Thellungiella halophile* in cotton improved shoot and root growth as compared to wild type. In addition, transgenic lines had higher chlorophyll content, improved photosynthesis and higher relative water content of leaves, and cell membrane damage was observed less than wild type. These properties improved root development and the lower solute potential resulting from higher solute content such as soluble sugars and free amino acids in the transgenic plants. These beneficial features enhanced drought tolerance in transgenic cotton, and seed cotton yield was 51% higher than wild‐type cotton plants (Lv *et al*., 2009). In another study, a gene, *ScALDH21,* from *Syntrichia carninervis* was transformed into cotton (Yang *et al*., 2016). Transgenic plants were checked in the green house as well as in the field for drought tolerance. Under field conditions, overexpressed transgenic lines showed greater plant height, larger bolls and greater fibre yield than wild type during different treatments of drought stress. It is due to improved proline and soluble sugars, greater photosynthetic rate and reduced lipid peroxidation in transgenic cotton as compared to wild type. These discoveries and other studies have led scientists to engineer drought‐tolerant plants (cotton) using genetic engineering methods, which are an effective technology at the present time. Thus, various transgenic plants, including cotton, have already been produced by inserting various stress‐related genes and then examined the plants for the specific traits, that is drought tolerance. However, field applications still need to be assessed because most of these experiments were performed in the laboratory and greenhouse conditions and did not produce appreciable results in the field.

**Table 4:** Successful stories of genetically modified cotton with enhanced yield under drought stress

Gene(s)	Promoter	Plant from which gene taken	Environmental condition	Abiotic stress type	Beneficial traits of transgenic cotton against drought stress	Effect on yield	References
*ScALDH21*	*CaMV 35S*	*Syntrichia caninervis*	Greenhouse and field	Drought	Soluble sugar and proline content increased, higher peroxidase activity, reduced loss of net photosynthetic rate, reduced lipid peroxidation, greater plant height, larger bolls	Yield increased	Yang *et al*. (2016)
*AtEDT1/HDG11*	*CaMV 35S*	*A. thaliana*	Laboratory Greenhouse and Field	Drought and salt	Soluble sugar and proline content increased, well‐developed roots, low stomatal density, increased ROS scavenging enzymes	43% higher seeds	Yu *et al*. (2015)
*SNAC1*	*CaMV 35S*	Rice	Greenhouse	Drought and salt	Enhanced proline content and root development, while transpiration rate decreased	131% more bolls	Liu *et al*. (2014)
*AVP1*	*CaMV 35S*	*A. thaliana*	Greenhouse and field	Drought and salt	Enhanced sequestration of ions and sugars into vacuole, reduced water potential, and enhanced root biomass	Increased 20%	Pasapula *et al*. (2011)
*Osmotin*	*CaMV 35S*	Tobacco	Greenhouse	Drought	Higher relative water content and proline level, while H_2_O_2_, lipid peroxidation, and electrolyte leakage were reduced	57.6% more bolls	Parkhi *et al*. (2009)
*TsVP*	*CaMV 35S*	*Thellungiella halophila*	Greenhouse	Drought	Improved root and shoot growth, higher rate of photosynthesis and relative water content, while less cell membrane damaged	42%–61% higher (Lumianyan 19) 27%–53% higher (Lumianyan 21)	Lv *et al*. (2009)
*betA*	*CaMV 35S*	*Eschercia coli*	Greenhouse	Drought	Increased photosynthesis, higher relative water content, better osmotic adjustment, less ion leakage and lipid membrane peroxidation	3%–12% higher	Lv *et al*. (2007)

### Exogenous application of substances

Exogenous application of osmoprotectants and various plant growth regulators have been found effectively to enhance drought tolerance in cotton. Foliar application of osmoprotectants and plant hormones, including ABA, gibberellic acid (GA_3_), salicylic acid (SA), proline, glycinebetaine and polyamines, has been reported to relieve the effects of stress. These treatments elevated osmotic adjustment to improve turgor pressure and promoted accumulation of antioxidants to detoxify ROS, thus maintaining the integrity of membrane structures, enzymes and other macromolecules during drought conditions (Anjum *et al*., 2011). For example, the exogenous application of proline and glycinebetaine as a foliar spray has also been found to be effective in reducing the adverse effects of drought stress on cotton (Noreen *et al*., 2013). In this way, GA exogenous application enhanced the net rate of photosynthesis, transpiration rate and stomata conductance in cotton (Lichtfouse *et al*., 2009). Similarly, Zhao *et al*. (2013) exogenously sprayed ABA, JA and MeJA on cotton plants. *GbRLK* was differentially induced by JA and MeJA, but it was gradually up‐regulated when exposed to ABA treatment.

## Concluding remarks and future perspectives

Currently, drought stress is responsible for extensive crop loss and will likely become worse in the future; thus, there is international interest in increasing drought‐tolerant crops. The goal of our study was to explore the mechanism of cotton under water‐deficit conditions. Scientists are attempting to induce drought tolerance in cotton as well as other important crops. The aim was to identify and enhance drought‐tolerant traits via QTL analysis, transgenic approaches and exogenous application of substances. Several genes have been identified and characterized by proteomic, transcriptomic and other omics that are induced by drought stress and the associated signalling and regulatory pathways in cotton plants. In comparison with *Arabidopsis*, the amount of data on drought‐regulated genes and their functions in cotton are inadequate. However, a few of these genes have been studied in cotton for their response to water‐deficit conditions, which is still in early stages. Transgenic cotton plants were mostly studied under greenhouse conditions or tested in the field under natural water‐deficit environments with a small amount (Table 4). It should be study in more realistic environment that is in the field that what really happens there. Usually, transgenic lines are developed by single gene transformation, which may not be as productive as if it had been developed by transferring a number of drought‐related genes. It seems interesting to transfer a number of prominent genes response to drought and yield in the same variety of cotton. The amount of data on drought‐associated cotton protein kinases is also limited. Only a few cotton protein kinases have been engineered and studied in *Arabidopsis* and tobacco (Table 2); however, there are no reports by engineering in cotton for enhancing drought tolerance. More work on cotton plants is needed, however, to link physiology, system biology and field performance. Understanding traits in cotton plants are associated with root architecture, stomatal conductance, photosynthesis and osmotic adjustment in drought stress. It is important to enhance the drought tolerance capability of cotton, and there is still much work to be performed to secure future generations from the upcoming crisis. Drought is a complex trait; however, rapid advances in the omics technologies will make it possible to use a system biology approach to understand cotton plants responses to drought stress and introduce drought‐tolerant cotton.
